# Kaempferia parviflora rhizome extract exerts anti-obesity effect in high-fat diet-induced obese C57BL/6N mice

**DOI:** 10.29219/fnr.v67.9413

**Published:** 2023-08-23

**Authors:** Hyun Sook Lee, Young Eun Jeon, Riyo Awa, Susumu Yoshino, Eun Ji Kim

**Affiliations:** 1 Department of Food Science & Nutrition, Dongseo University, Busan, Korea; 2 Industry Coupled Cooperation Center for Bio Healthcare Materials, Hallym University, Chuncheon, Korea; 3 Research Center, Maruzen Pharmaceuticals Co. Ltd., Hiroshima, Japan

**Keywords:** Kaempferia parviflora, *anti-obesity*, *adipogenic transcription factors*, *adipokines*, *C57BL/6N mice*

## Abstract

*Kaempferia parviflora* (KP) rhizome, also called black ginger, has been used as a herbal medicine for many centuries. This current study was aimed at exploring whether KP rhizome extract (KPE) had anti-obesity effects and the mechanism involved. Five-week-old C57BL/6N male mice were allocated into five groups for 8-week feeding with control diet (CD), high-fat diet (HFD), HFD + 150 mg/kg body weight (BW)/day KPE (HFD+K150), HFD + 300 mg/kg BW/day KPE (HFD+K300), and HFD + 600 mg/kg BW/day KPE (HFD+K600). KPE decreased BW, body fat mass, adipose tissue weight, adipocyte size, and serum levels of glucose, triglycerides, cholesterol, insulin, and leptin in HFD-induced obese C57BL/6N mice. KPE inhibited adipogenesis by decreasing CCAAT/enhancer binding protein α, peroxisome proliferator-activated receptor γ, sterol regulatory element-binding protein-1c, acetyl-CoA carboxylase 1, ATP-citrate lyase, and fatty acid synthase mRNA expression. KPE improved lipolysis by increasing carnitine palmitoyl transferase 1 and hormone-sensitive lipase mRNA expression. These results suggest that KPE may have inhibited HFD-induced obesity by regulating several pathways involved in decreasing adipogenesis and enhancing lipolysis. Thus, the results suggest that KPE (or KP) may be applicable as an anti-obesity agent.

## Popular scientific summary

- Treatment with Kaempferia parviflora rhizome extract decreased body weight, body fat mass, adipose tissue weight, adipocyte size, and serum levels of glucose, triglyceride, cholesterol, insulin, and leptin in HFD-induced obese C57BL/6N mice.
- Treatment with Kaempferia parviflora rhizome extract inhibited HFD-induced obesity by regulating several pathways involved in decreasing adipogenesis and enhancing lipolysis in adipose tissue.

Globally, obesity is considered an increasingly important health problem. Obesity is a state of weight gain due to the excessive accumulation of body fat, which is a major risk factor for various diseases, such as hyperlipidemia, diabetes, hypertension, gout, and osteoporosis. As the degree of obesity increases, the incidence of serious diseases, such as cardiovascular diseases and cancer, increases, and treatment becomes more difficult (1–3). Since obesity is closely related to various diseases, strategies to prevent and improve obesity have a very important impact in ameliorating these diet-related diseases. Recently, a wide variety of functional foods or natural components have been developed for improving or preventing obesity. Plant-based therapy or supplementation is safe, has various physiological activities and almost no side effects compared to drugs even when taken for a long time, and is relatively inexpensive. Thus, it is actively considered in the treatment of various diseases (4). In the case of obesity, particularly, it is rare to find drugs approved for long-term use, so there is a high interest in research on plant materials that can replace existing drugs or be developed as new pharmaceutical agents (5).

*Kaempferia parviflora* (KP) rhizome, or black ginger, is an herbaceous plant belonging to the *Zingiberaceae* family and is native to tropical countries. In Thailand, the rhizome of KP is called Kurachai Dum and has been used as a traditional remedy to decrease blood glucose levels, improve blood circulation, and boost vitality. KP rhizome extract has been reported to have various pharmacological effects, including anti-inflammatory (6), antioxidant (7), anti-peptic ulcer (8), anticancer (9), cardioprotective (10), anti-obesity (11, 12), anti-osteoarthritis (13), neuroprotective (14), and antidiabetic (15) activities. It was also shown to induce skeletal muscle hypertrophy (16) and enhance endurance capacity (17). KP rhizome contains numerous active constituents, such as alkaloid, glycoside, flavonoid, phenol, polyphenol, lipophenol, saponin, tannin, terpene, and steroid components (18). In particular, it contains several kinds of flavonoids and flavonoid glycosides with abundant methoxy groups. Among them, polymethoxyflavones (PMF), including 3,5,7,3’,4’-pentamethoxyflavone and 5,7-dimethoxyflavone (DMF), are known to possess the main pharmacological efficacy of KP rhizome (19–21). The effect of KP extract on the accumulation of fat in tissues varies depending on differences in extraction solvents and the resulting chemical structure of the extract (22). Ethyl acetate and ethanol extracts of KP rhizome were reported to have great anti-obesity effects (23). The anti-obesity effect of KP rhizome extract was reported in several experimental animals, including obese rats induced by high-fat diet (HFD) (24) and high-fat and high-sugar diet (25), and in type 2 diabetes model mice (23). The oral administration of KP rhizome extract was also reported to have an anti-obesity effect in healthy (24, 26) and obese people (27).

These results showed that KP rhizome had the effect of increasing energy consumption and fat utilization not only *in vitro* and animals but also humans and has the potential to be used as an anti-obesity agent. However, KP is less widely known than other plants with anti-obesity properties (4), and the mechanism of KP’s anti-obesity effect has not been fully examined. This study was conducted to examine the effects of KP rhizome extract on improving obesity and body fat and obesity-related genes. Among KP extraction solvents, ethanol is known to extract many active ingredients that are most effective in improving obesity, so was used in this study. Then, it was orally administered to C57BL/6N mice fed HFD to induce obesity.

## Materials and methods

### Preparation of Kaempferia parviflora extract

*Kaempferia parviflora* rhizome extract (KPE) was prepared by Maruzen Pharmaceuticals Co., Ltd (Hiroshima, Japan). In brief, dried KP rhizome was extracted with 60% ethanol. After extraction, concentration was carried out under reduced pressure, and after mixing the extract with an equal amount of dextrin and γ-cyclodextrin, KPE powder was obtained by spray-drying. The identification and quantification of 3,5,7,3’,4’-pentamethoxyflavone in KPE were performed by high-performance liquid chromatography. The 3,5,7,3’,4’-pentamethoxyflavone content in KPE was 2.6% (w/w) (Lot. FD-2022).

### Animals and ethical statement

Four-week-old male C57BL/6N mice were obtained by Dooyeol Biotech Co. Ltd. (Seoul, Korea) and were kept at the animal research facility of Hallym University. The animal facility was maintained at 23 ± 3°C and 50 ± 10% relative humidity, with a 12-h light (8 am to 8 pm)/dark cycle. The mice were adapted to the environment for 1 week prior to experimentation. During the adaptation period, the mice were allowed free access to a commercial, non-purified rodent diet and tap water.

The animal study protocol was approved by the Institutional Animal Care and Use Committee of Hallym University (approved number: Hallym 2022-10).

### Experimental design

After a 1-week adaptation period, the mice were randomly divided into five groups (*n* = 10 per group) as follows: 1) control diet (CD), 2) HFD, 3) HFD + 150 mg/kg body weight (BW)/day KPE (HFD+K150), 4) HFD + 300 mg/kg BW/day KPE (HFD+K300), and 5) HFD + 600 mg/kg BW/day KPE (HFD+K600) group. The mice in the CD group were fed a CD (with 10% kcal from fat, 20% kcal from protein, and 70% kcal from carbohydrates; Cat. no. D124505B, Research Diets, Inc., New Brunswick, NJ, USA), while the mice in the other groups were fed a HFD (with 60% kcal from fat, 20% kcal from protein, and 20% kcal from carbohydrates; Cat. no. D12452, Research Diets, Inc.). Food and water were fed *ab libitum* during the entire experimental period. KPE dissolved in sterile distilled water was given orally every day by gavage for 8 weeks. An equal volume of sterile distilled water was administered orally to the mice in the CD and HFD groups at the same time. Food intake and BW were determined daily and once a week, respectively, during the entire experimental period.

At the end of the experimental period, the mice were anesthetized with tribromoethanol diluted in amyl alcohol, following a 16-h fast. Blood was subsequently taken from the orbital vein, and serum was separated from the blood by centrifuging it at 3,000 rpm for 20 min at 4°C. The mice were euthanized by cervical dislocation after blood was collected, and four separate areas of white adipose tissue (WAT, epididymal, retroperitoneal, mesenteric, and inguinal) were quickly excised, rinsed with physiological saline, and weighed.

### Body fat mass assessment

One day prior to the termination of the experiment, body mass percentage was determined using dual-energy X-ray absorptiometry (DEXA, PIXImus^TM^, GE Lunar, Madison, WI, USA).

### Serum biochemical analysis

Glucose, triglycerides (TG), total cholesterol, low-density (LDL)-cholesterol, and high-density (HDL)-cholesterol levels in serum were measured by a blood chemistry autoanalyzer (KoneLab 20XT, Thermo Fisher Scientific, Vantaa, Finland). Serum insulin levels were determined using an enzyme-linked immunosorbent assay (ELISA) kit (Millipore, Billerica, MA, USA). The serum levels of leptin and adiponectin were measured using the appropriate ELISA kit (R&D Systems, Minneapolis, MN, USA), according to the manufacturer’s instructions. Homeostasis model assessment of insulin resistance (HOMA-IR) was calculated by the following formula: [fasting glucose (mg/dL) × fasting insulin (mU/L)]/405 (28). The quantitative insulin sensitivity check index (QUICKI) was calculated by the following formula: 1/[log fasting glucose (mg/dL) + log fasting insulin(mU/L)] (29).

### Histological analysis

Epididymal adipose tissues were fixed with 4% paraformaldehyde in phosphate buffer (0.5 M, pH 7.4), embedded in paraffin, and cut to a thickness of 5 μm. Then, the tissue sections were stained with hematoxylin and eosin (H&E), examined, and photographed under a light microscope (AxioImager, Carl Zeiss, Jena, Germany) at 200× magnification. Adipocyte size was analyzed using the AxioVision Imaging System (Carl Zeiss).

### Quantitative reverse transcription-polymerase chain reaction

Total RNA was extracted from mesenteric adipose tissue, and real-time reverse transcription-polymerase chain reaction (RT-PCR) was performed using a Rotor-Gene^TM^ SYBR Green kit (Qiagen, Valencia, CA, USA) and a Rotor-gene 3000 PCR machine (Corbett Research, Mortlake, Australia) (30). The sequences of the PCR primers used in this study are listed in Table 1. The results were analyzed using the Rotor-Gene 6000 Series system software, version 6 (Corbett Research), and the relative expression of the target genes was normalized to that of glyceraldehyde-3-phosphate dehydrogenase (*GAPDH*).

**Table 1:** Primer sequences used in this study

Target gene	Forward primer (5’-3’)	Reverse primer (5’-3’)
ACC1	GGAGATGTACGCTGACCGAGAA	ACCCGACGCATGGTTTTCA
ACL	TGGATGCCACAGCTGACTAC	GGTTCAGCAAGGTCAGCTTC
C/EBPα	TGGACAAGAACAGCAACGAGTAC	GCAGTTGCCCATGGCCTTGAC
CPT1	CCTGGAAGAAACGCCTGATT	CAGGGTTTGGCGAAAGAAGA
FAS	AGGGGTCGACCTGGTCCTCA	GCCATGCCCAGAGGGTGGTT
HSL	CCGTTCCTGCAGACTCTCTC	CCACGCAACTCTGGGTCTAT
PPARγ	CAAAACACCAGTGTGAATTA	ACCATGGTAATTTCTTGTGA
SREBP-1c	CACTTCTGGAGACATCGCAAAC	ATGGTAGACAACAGCCGCATC
GAPDH	TGGGTGTGAACCATGAGAAG	GCTAAGCAGTTGGTGGTGC

### Statistical analysis

All data are expressed as the mean ± standard error of the mean (SEM). SAS for Windows version 9.1 (SAS Institute, Cary, NC, USA) was used to conduct statistical analyses. Student’s *t*-test was used to test the difference between the CD and HFD groups. Analysis of variance followed by Duncan’s multiple comparison test was used to compare means among the HFD, HFD+K150, HFD+K300, and HFD+K600 groups. *P* < 0.05 was considered significant.

## Results

### KPE reduces BW gain

**Fig. 1:**
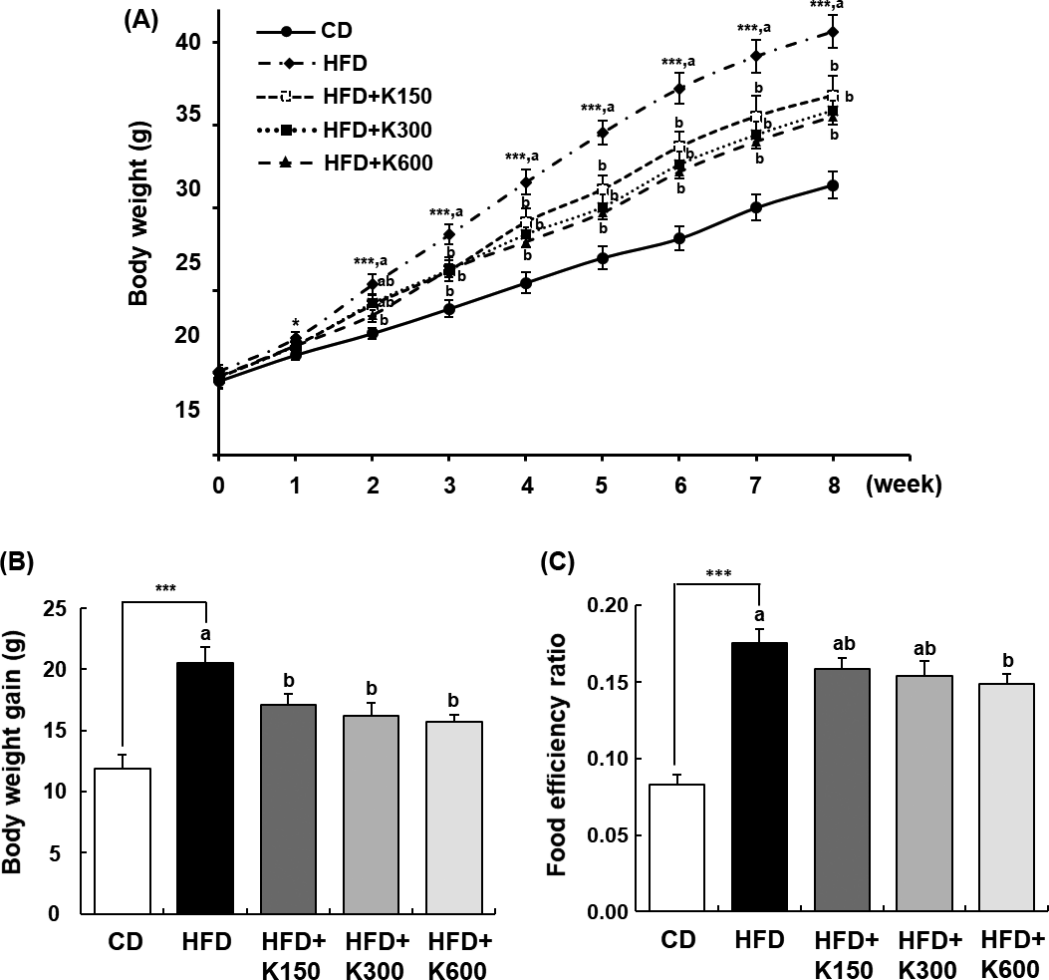
Effect of *Kaempferia parviflora* rhizome extract (KPE) treatment on body weight change, body weight gain, and food efficiency ratio in high-fat diet (HFD)-fed C57BL/6N mice. Mice fed with HFD were treated with KPE by oral gavage for 8 weeks. Body weights were measured every week. The food efficiency ratio was calculated by the following formula: [weight gain (g)/food intake (g)]. (A) Body weight. (B) Body weight gain. (C) Food efficiency ratio. Values are expressed as the mean ± SEM (*n* = 10). ^*^P < 0.05, ^**^P < 0.01, and ^***^P < 0.001 significantly different from the CD group. Different letters indicate significant difference among the HFD, HFD+K150, HFD+K300, and HFD+K600 groups at *P* < 0.05.

The BW of mice in the HFD group was significantly higher than that in the CD group 1 week after the start of the experiment. In contrast, the BW of the HFD+K600 group was significantly lower than that of the HFD group from the second week and the HFD+K150 and HFD+K300 groups from the third week. These effects continued until the end of the experiment (Fig. 1A). KPE inhibited HFD-induced weight gain. After the end of the 8-week experiment, the BW gains of mice in the HFD+K150, HFD+K300, and HFD+K600 groups decreased by 16.7, 21.0, and 23.4%, respectively, compared to the HFD group (Fig. 1B). The food efficiency ratio was significantly increased in the HFD group compared to the CD group (*P* < 0.001), and it was significantly decreased in the HFD+K600 group (*P* < 0.05, Fig. 1C).

### KPE reduces body fat deposition

**Fig. 2:**
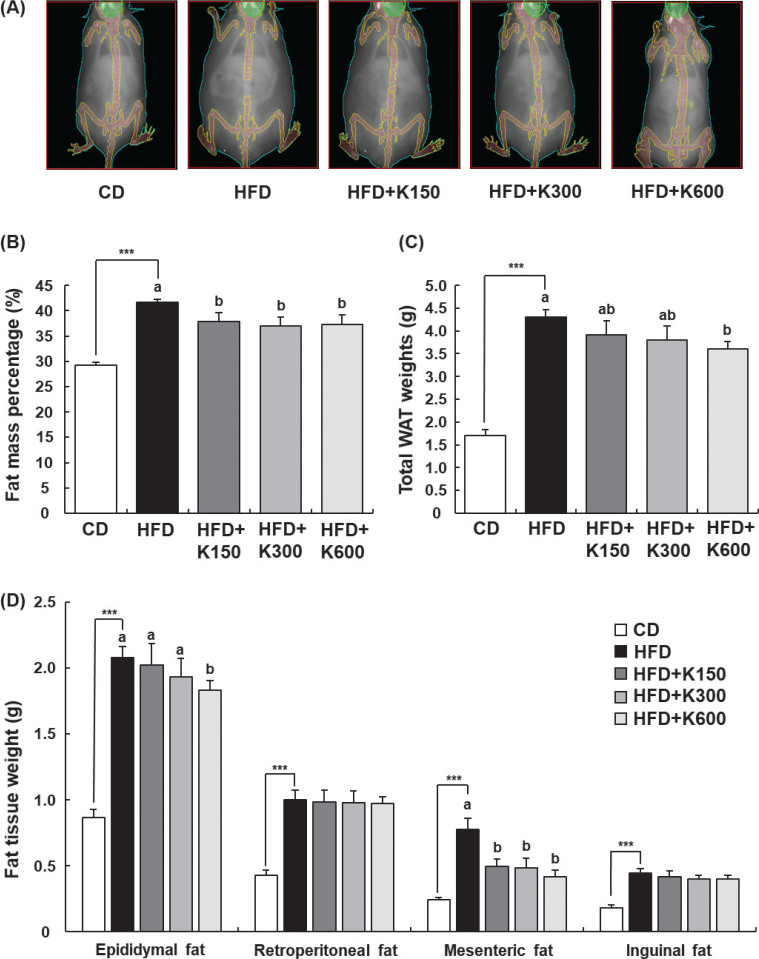
Effect of KPE treatment on adipose tissue weight in HFD-fed HFD C57BL/6N mice. Mice fed with HFD were treated with KPE by oral gavage for 8 weeks. The body mass percentage was measured using DEXA. WAT (epididymal, retroperitoneal, mesenteric, and inguinal) was excised and weighed. (A) Representative DEXA images of whole body fat. (B) Body fat mass percentage measured by DEXA. (C) Total WAT weights calculated as the sum of epididymal, retroperitoneal, mesenteric, and inguinal fat. (D) Adipose tissue weight in epididymal, retroperitoneal, mesenteric, and inguinal fat. Values are expressed as mean ± SEM (*n* = 10). ^***^P < 0.001 significantly different from the CD group. Different letters indicate significant difference among the HFD, HFD+K150, HFD+K300, and HFD+K600 groups at *P* < 0.05.

Body fat mass analyzed by DEXA was significantly increased by HFD (*P* < 0.001), which was inhibited by KPE treatment. Fat mass percentage in the HFD+K600 group decreased by 10.6% compared to the HFD group (Fig. 2A and B). Total WAT was increased significantly in the HFD group compared with the CD group and decreased significantly in the HFD+K600 group compared with the HFD group (*P* < 0.05, Fig. 2C). Regarding the adipose tissue in each compartment, increases in fat deposition in epididymal fat pads and mesenteric fat caused by HFD showed a tendency to be prevented by KPE treatment. However, there was no effect of KPE treatment on retroperitoneal fat and inguinal fat (Fig. 2D).

**Fig. 3:**
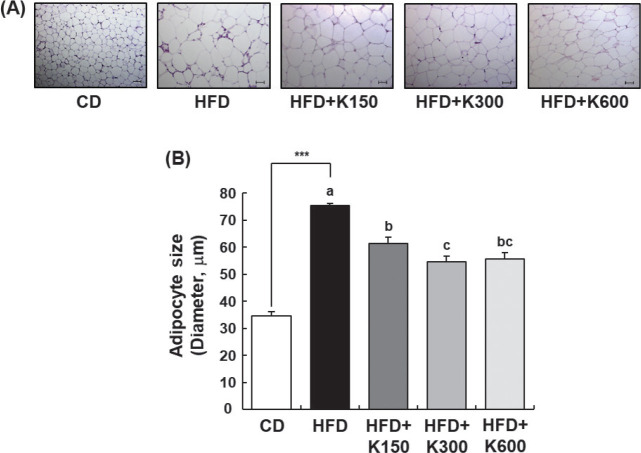
Effect of KPE treatment on morphological changes in epididymal adipose tissue in HFD-fed C57BL/6N mice. Mice fed with HFD were treated with KPE by oral gavage for 8 weeks. The extracted epididymal adipose tissues were fixed, embedded in paraffin, and cut at 5 μm. Tissue sections were stained with H&E. The size of the adipocytes was quantified by measuring the longest diameter of the adipocytes. (A) Representative H&E-stained images of epididymal adipose tissue (*n* = 5), 200× magnification, scale bar = 50 μm. (B) Adipocyte size. Values are expressed as mean ± SEM (*n* = 10). ***P < 0.001 significantly different from the CD group. Different letters indicate significant difference among the HFD, HFD+K150, HFD+K300, and HFD+K600 groups at *P* < 0.05.

KPE significantly decreased adipocyte size (Fig. 3). The histological analysis of epididymal fat showed that the size of adipocytes in epididymal fat pads in the HFD group was significantly larger than that in the CD group and was reduced by KPE treatment (*P* < 0.001). However, there was no difference according to KPE dose (Fig. 3A and B). These results show that the oral administration of KPE reduced adipocyte size and fat volume, leading to a significant decrease in fat accumulation.

### KPE improves insulin resistance and serum lipid profiles

Blood glucose concentrations were significantly increased in the HFD group compared with the CD group (*P* < 0.01). The serum glucose levels in the HFD group were not significantly different from those in the HFD+K150 and HFD+K300 groups and were significantly decreased only in the HFD+KP600 group (*P* < 0.05). Serum insulin concentrations were significantly increased in the HFD group compared with the CD group (*P* < 0.001) and were significantly decreased by KPE treatment in the HFD+K150 and HFD+K600 groups. To determine whether KPE suppressed insulin resistance, HOMA-IR and QUICKI were measured. HOMA-IR was increased significantly in the HFD group compared with the CD group (*P* < 0.001) and decreased by KPE treatment in the HFD+K150 and HFD+K600 groups. QUICKI was decreased in the HFD group compared with the CD group (*P* < 0.001) and increased by KPE treatment in the HFD+K150 and HFD+K600 groups. HOMA-IR and QUICKI levels in the HFD+K300 group were not significantly different from those in the HFD group, but the HFD+K150 and HFD+K600 groups showed a significant difference compared to the HFD group, and the effect was greater in the latter than in the former (Table 2).

**Table 2:** Effect of *Kaempferia parviflora* extract on serum levels of glucose, lipids, insulin, leptin, and adiponectin in HFD-fed C57BL/6 mice

Variables	CD	HFD	K150	K300	K600
Glucose (mg/dL)	97.7 ± 15.7	151.5 ± 7.3^**,ab^	138.3 ± 8.2^bc^	165.3 ± 6.6^a^	123.1 ± 9.6^c^
Triglyceride (mg/dL)	71.9 ± 3.9	101.7 ± 8.0^**,a^	88.2 ± 5.2^ab^	69.0 ± 4.1^c^	80.6 ± 5.8^c^
Total cholesterol (mg/dL)	135.5 ± 11.1	195.7 ± 8.7^***,a^	179.0 ± 14.0^ab^	172.6 ± 7.0^ab^	163.0 ± 9.5^b^
LDL-cholesterol (mg/dL)	32.2 ± 1.6	42.7 ± 1.8^***,a^	39.1 ± 4.3^a^	37.2 ± 2.3^ab^	35.6 ± 2.5^b^
HDL-cholesterol (mg/dL)	123.0 ± 14.5	188.5 ± 8.6^**,a^	164.6 ± 13.5^ab^	162.6 ± 6.5^ab^	151.7 ± 9.2^b^
Insulin (ng/mL)	2.69 ± 0.26	7.61 ± 0.52^***,a^	5.43 ± 0.87^b^	5.64 ± 0.69^ab^	4.49 ± 0.66^b^
HOMA-IR1	19.24 ± 3.26	68.47 ± 5.32^***,a^	46.07 ± 8.09^b^	56.32 ± 7.71^ab^	37.20 ± 7.62^b^
QUICKI2	0.264 ± 0.008	0.226 ± 0.002^***,b^	0.240 ± 0.006^a^	0.232 ± 0.003^ab^	0.245 ± 0.005^a^
Leptin (ng/mL)	4.46 ± 0.56	21.41 ± 1.93^***,a^	12.10 ± 1.10^b^	10.64 ± 1.18^b^	9.25 ± 1.05^b^
Adiponectin (mg/dL)	24.64 ± 1.15	20.99 ± 0.97^*^	20.82 ± 1.09	20.68 ± 1.48	19.57 ± 0.84

^1^Homeostasis model assessment of insulin resistance (HOMA-IR) was calculated on the basis of the formula: [fasting glucose (mg/dL) × fasting insulin (mU/L)]/405.

^2^Quantitative insulin sensitivity check index (QUICKI) was calculated on the basis of the formula: [1/[log fasting glucose (mg/dL) + log fasting insulin (mU/L)]. Values are expressed as the mean ± SEM (*n* = 10).

* *P* < 0.05,

** *P* < 0.01, and

*** *P* < 0.001 significantly different from the CD group.

Different letters indicate significant differences among the HFD, HFD+K150, HFD+K300, and HFD+K600 groups at *P* < 0.05.

Blood TG and cholesterol levels were significantly elevated in the HFD group compared with the CD group (*P* < 0.01 and *P* < 0.001, respectively). Serum TG levels were significantly lower in the HFD+K300 and HFD+K600 groups than in the HFD group. Serum total cholesterol and LDL-cholesterol levels were significantly lower in the HFD+K600 group than in the HFD group. HDL-cholesterol levels were increased significantly in the HFD group compared with the CD group (*P* < 0.01) and tended to decrease in proportion to the KPE dose (Table 2).

### KPE decreased leptin levels

The HFD group showed a significant increase in leptin concentrations compared with the CD group (*P* < 0.001). The levels were significantly reduced by KPE treatment. The leptin levels in the HFD+K150, HFD+K300, and HFD+K600 groups were decreased by 43.5, 50.3, and 56.8%, respectively, compared with the HFD group. Adiponectin levels were decreased significantly in the HFD group compared with the CD group (*P* < 0.05). Serum adiponectin levels were not affected by KPE treatment (Table 2).

### KPE modulates the expression of genes related to lipid metabolism in mesenteric adipose tissue

**Fig. 4:**
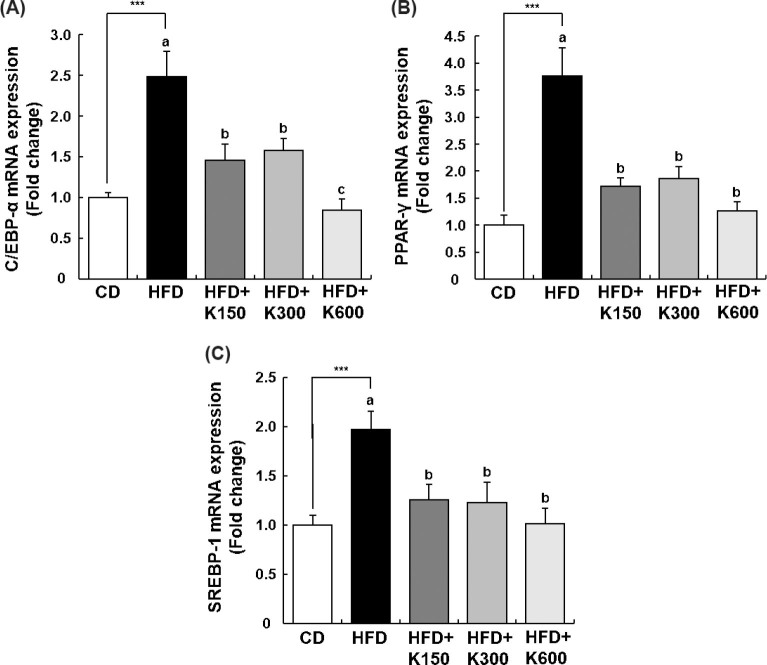
Effect of KPE treatment on the expression of adipogenic transcription factors in the mesenteric adipose tissue of HFD-fed C57BL/6N mice. Mice fed with HFD were treated with KPE by oral gavage for 8 weeks. The total RNA in mesenteric adipose tissue was isolated and reverse transcribed, and a real-time PCR was conducted. The expression of *C/EBPα* (A), *PPARγ* (B), and *SREBP-1c* (C) mRNA was normalized to the expression of *GAPDH* mRNA and presented relative to the CD group. Values are expressed as mean ± SEM (*n* = 10). ^***^P < 0.001 significantly different from the CD group. Different letters indicate significant difference among the HFD, HFD+K150, HFD+K300, and HFD+K600 groups at *P* < 0.05.

We observed that KPE decreased the weight of mesenteric fat tissue most significantly (Fig. 2D). Thus, we analyzed the mRNA expression of adipogenesis-related transcription factors and their target genes in mesenteric adipose tissues to investigate the molecular mechanism of KPE’s anti-obesity effect. The mRNA expression of CCAAT/enhancer binding protein α (*C/EBPα*), peroxisome proliferator-activated receptor γ (*PPARγ*), and sterol regulatory element binding protein-1c (*SREBP-1c*) were notably increased in the HFD group compared with the CD group (*P* < 0.001, *P* < 0.001, and *P* < 0.001, respectively). Increases in mRNA expression of *C/EBPα, PPARγ*, and *SREBP-1c* were significantly prevented by KPE treatment (Fig. 4). The mRNA expression of *C/EBPα*, *PPARγ*, and *SREBP-1c* was decreased more in the HFD+K600 group than in the HFD+K150 and HFD+K300 groups. There was no statistically significant difference between the HFD+K150 and HFD+K300 groups (Fig. 4).

**Fig. 5:**
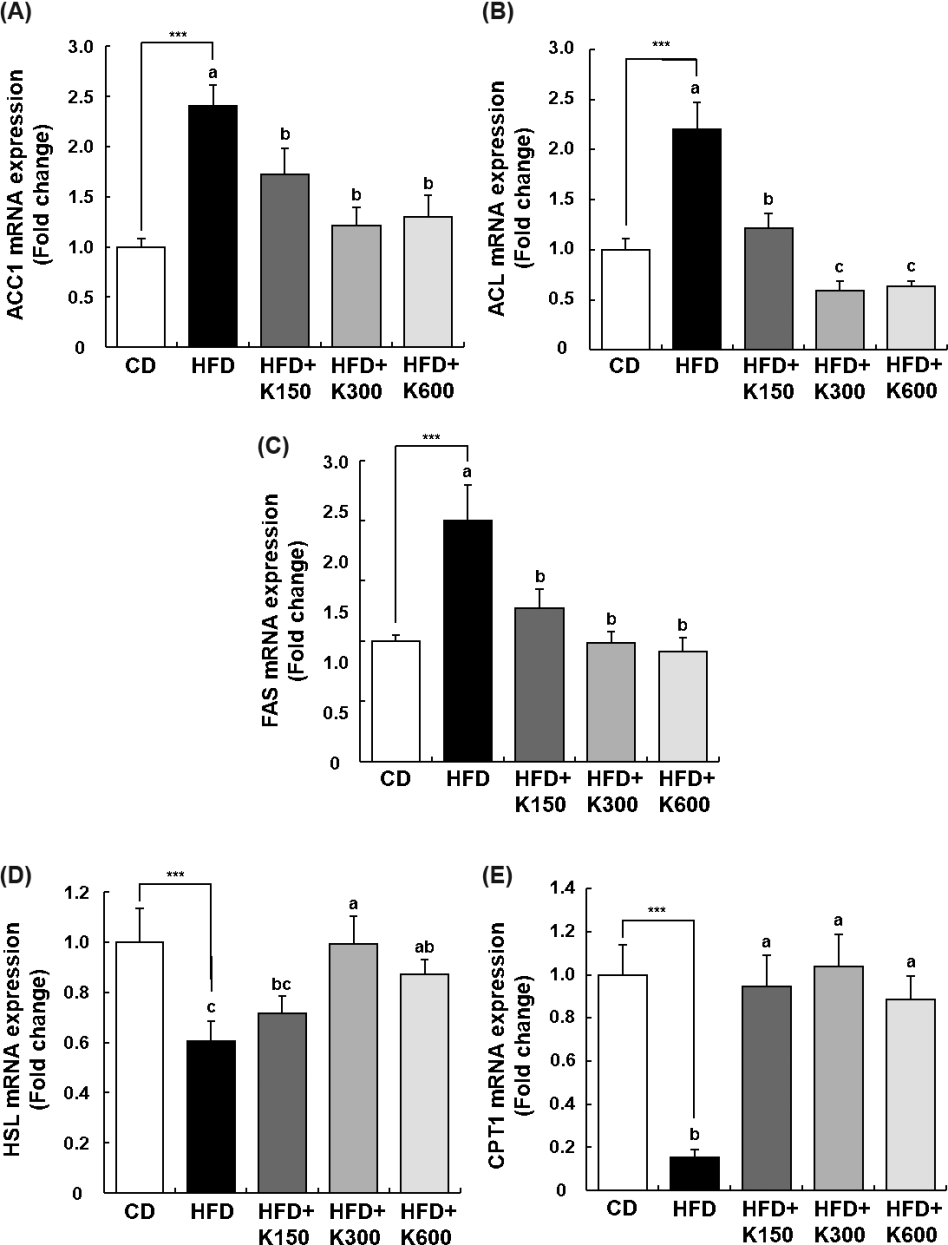
Effect of KPE treatment on the expression of adipogenesis- and lipolysis-related genes in the mesenteric adipose tissue of HFD-fed C57BL/6N mice. Mice fed with HFD were treated with KPE by oral gavage for 8 weeks. The total RNA in mesenteric adipose tissue was isolated and reverse transcribed, and a real-time PCR was conducted. The expression of *ACC1* (A), *ACL* (B), *FAS* (C), *HSL* (D), and *CPT1* (E) mRNA was normalized to the expression of *GAPDH* mRNA and presented relative to the CD group. Values are expressed as mean ± SEM (*n* = 10). ^***^P < 0.001 significantly different from the CD group. Different letters indicate significant difference among the HFD, HFD+K150, HFD+K300, and HFD+K600 groups at *P* < 0.05.

The mRNA expression of acetyl-CoA carboxylase 1 (ACC1), ATP citrate lyase (ACL), and fatty acid synthase (FAS) genes related to adipogenesis was analyzed. The mRNA expressions of ACC1, ACL, and FAS were markedly increased in the HFD group compared with the CD group (*P* < 0.001). KPE treatment significantly decreased the mRNA expression of ACC1, ACL, and FAS. However, the decrease was not completely proportional to the dose. In the case of ACC1, the HFD+K300 group showed better results than the HFD+K600 group, and no significant difference in ACL and FAS was detected between the HFD+K300 and HFD+ K600 groups (Fig. 5A–C).

HSL and CPT1, which are enzymes related to lipolysis or fatty acid oxidation, were analyzed. The mRNA expression of *HSL* and *CPT1* was significantly decreased in the HFD group compared with the CD group, and KPE treatment increased the mRNA expressions of HSL and CPT1 decreased by HFD to the same level as in the CD group. There was no difference according to KPE dose (Fig. 5D and E).

## Discussion

An anti-obesity effect of KPE supplementation was reported in genetically obese and/or type II diabetes animal models (23, 31, 32), dietary induced obese animal model (25, 33), and also overweight and preobese humans (12). In ob/ob mice, a genetically obese model (32), and Tsumura, Suzuki, Obese Diabetes (TSOD) mice, a spontaneous obese type II diabetes model (34), KPE effectively inhibited obesity by inhibiting fat accumulation, hyperinsulinemia, glucose intolerance, hypertension, and insulin resistance. In our study, an anti-obesity effect of KPE was also confirmed. The KPE-treated groups demonstrated a decrease in BW gain, body fat mass, adipose tissue weight, adipocyte size, and serum levels of glucose, insulin, TG, cholesterol, and leptin. Several recent studies demonstrated that KPE activates brown adipose tissue and increases energy expenditure, resulting in a preventive effect on obesity (11, 22, 26). In addition to this, studies to reveal the mechanism of the anti-obesity effect of KPE are being actively conducted recently.

In our study, mesenteric fat and retroperitoneal fat mass were measured among visceral fat, and KPE particularly reduced mesenteric fat mass. Mesenteric fat is anatomically directly connected to the intestinal serosa and muscularis propria and surrounds most of the small and large intestines continuously along the axis. Mesenteric fat exhibits metabolic characteristics dissimilar from other abdominal fat deposits, such as subcutaneous and extraperitoneal fat (34). This may account for the closer connection between mesenteric fat thickness and several metabolic derangements in obese subjects. Among visceral fat, mesenteric fat is closely related to various inflammatory diseases and metabolic syndrome. Therefore, it is speculated that the reduction in mesenteric fat by KPE may contribute to preventing obesity-related complications in addition to ameliorating simple obesity.

Adipose tissue not only stores fat but also performs a cell regulatory function using a complex network of endo, para, and autocrine signals. The hypertrophy and hyperplasia of adipocytes are hallmarks of obesity, leading to increased leptin and decreased adiponectin secretion. These also induce insulin resistance and increase the risk of type 2 diabetes. Leptin is secreted from WAT, and its most important function is the regulation of energy homeostasis and metabolism. Blood leptin levels are positively correlated with obesity and weight gain. When fat cells increase, leptin levels increase proportionately and then bind and signal the brain’s leptin receptor (LEP-R) to suppress food intake and increase energy expenditure. However, as the positive energy balance continues and BW increases, leptin resistance develops. Leptin resistance decreases satiety and increases nutrient consumption. In other words, excessive leptin concentration causes obesity (35). Insulin is the primary regulator of leptin production. In the presence of prolonged hyperinsulinemia, plasma leptin concentrations increase. Insulin is speculated to stimulate leptin production through glucose metabolism (35). Adiponectin, a more abundant adipocyte-specific adipokine, has anti-inflammatory and insulin sensitivity-modulating effects. Adiponectin regulates glucose and lipid metabolism by promoting fatty acid oxidation in major target tissues, including skeletal muscle, liver, and adipose tissue. The production of adiponectin in adipocytes decreases as fat accumulates in lipid droplets after differentiation. In this study, KPE was very effective in preventing elevations in plasma leptin levels caused by a HFD. However, KPE did not prevent reductions in adiponectin concentrations caused by a HFD. These results are different from previous studies in which KP decreased leptin and increased adiponectin in obese or HFD animal models (11, 31, 36, 37). The immune system is strongly influenced by leptin’s strong pro-inflammatory effects, and leptin can be secreted by immune stimuli, such as interleukin (IL)-1, IL-6, lipopolysaccharide (LPS), or bacterial infection. Especially in obese people, leptin acts as a proinflammatory adipokine and is related to inflammatory diseases (38).

Since obesity is linked to chronic inflammatory processes, it is very encouraging that KPE effectively prevented HFD-induced increases in leptin concentrations in our study. Meanwhile, unlike other previous studies, the reason why KPE did not increase serum adiponectin levels may be due to various reasons, such as differences in active ingredients according to the extraction method. This may be because KPE has a greater effect on inhibiting the differentiation of preadipocytes than on adipocyte hypertrophy during adipogenesis. Thus, additional studies, such as adipokine gene expression analysis by KPE treatment, are needed.

Adipogenesis is a complex process in a series of stages, by which the differentiation of precursor cells, mainly stem cells-drove, to mature adipocytes is mediated by numerous transcription factors, cell-cycle proteins, hormones, and small molecules (4). Normal adipose tissue consists of small adipocytes, which are differentiated from preadipocytes. Therefore, the regulation of preadipocyte differentiation and adipocyte hypertrophy is useful strategies for preventing obesity (31). *C/EBP* family and *PPAR*γ play critical roles in adipogenesis. When hormonal signals are stimulated during adipogenesis, C/EBP is quickly activated and serves as a transcriptional regulator of *PPARγ. PPARγ* acts as the master regulator of adipogenesis (39). *PPARγ*, which belongs to the nuclear receptor family, is a ligand-regulated transcription factor that is primarily expressed in adipose tissue and controls the transcription of several genes in both white and brown adipocytes (40). *SREBP-1* plays a key role in the induction of lipogenesis by the liver. Insulin stimulates the expression of *SREBP-1c*, which controls the genes that are necessary for the production of fatty acids, lipids, and genes involved in glucose metabolism. Insulin-stimulated *SREBP-1c* enhances lipogenesis (conversion of carbohydrates into fatty acids) and glycolysis by activating the glucokinase enzyme (41). Lee et al. (32) reported that in the epididymal fat of ob/ob mice, KPE suppressed adipogenic transcription factor (PPARγ) and lipogenic enzymes (lipoprotein lipase, ACC1, and FAS) by upregulating AMP-activated protein kinase (AMPK). Song et al. (42) reported that KP attenuates obesity by downregulating the mRNA expression of PPARγ and C/EBPβ in high-fat-diet-induced C57BL/6J mice.

In adipocytes isolated from mesenteric adipose tissue, we investigated whether *C/EBPα*, *PPARγ*, and *SREBP-1c*, which are major transcription factors in adipogenesis and lipogenesis, including preadipocyte differentiation and lipid biosynthesis, were affected by KPE. The HFD group demonstrated significantly higher *C/EBPα, SREBP-1c*, and *PPARγ* levels compared with the CD group. Treatment with KPE substantially reduced the mRNA expression levels of *PPARγ*, *SREBP-1c*, and *C/EBPα*. Additionally, the mRNA expression of adipogenesis-related genes, *ACC1*, *ACL*, and *FAS*, which are affected by these transcription factors, was increased nearly 2.5-fold by HFD but decreased dramatically by KPE treatment to almost the same level as that in the CD group. In contrast, *CPT1* and *HSL*, lipolysis-related genes affected by the above transcription factors, were significantly decreased by HFD but recovered to the same level as the CD group by KPE. These results demonstrate at the molecular level that KPE affected adipogenesis-related transcript factors and genes, inhibited adipogenesis, and promoted lipolysis.

Regarding safety, we analyzed the blood concentrations of aspartate aminotransferase (AST) and alanine aminotransferase (ALT) as markers of liver toxicity in the KPE-administered groups, but there was no significant difference between the experimental groups. Also, there were no anatomical changes in the liver (data not shown). The concentration of KPE we used was 0.375–1.5 g/day. According to Saokaew et al. (43), who systematically reviewed the clinical effects of KPE, no adverse effects were reported even at 1.35 g/day, and no adverse reactions from KPE administration have been reported yet. In animal studies related to the chronic toxicity of KPE, KPE was orally administered to Wistar rats at doses of 5, 50, and 500 mg/kg/day for 6 months. These doses are 1, 10, and 100 times, respectively, the amount used in humans. Even in these conditions, KPE did not show any toxicity (44). Therefore, it seems that KPE can be used as a relatively safe agent for obesity treatment.

## Conclusions

In this research, we verified that KPE exerted an anti-obesity effect on HFD-induced obese C57BL/6N mice. Leptin resistance, insulin resistance, dyslipidemia, adipocyte size, BW, fat mass, and adipose tissue weight were all lower in mice treated with KPE. As for the molecular mechanism of these changes, KPE reduced adipogenesis-related transcription factors (*C/EBPα*, *SREBP-1c*, and *PPARγ*) and changed transcription factors that affect genes related to them. This can be partially explained by the downregulation of specific genes associated with adipogenesis (*ACC1*, *ACL*, and *FAS*) and the upregulation of specific genes related to lipolysis (*CPT1* and *HSL*). Our findings suggest that KPE (or KP) is likely to be an anti-obesity agent candidate.

## Data Availability

Data available on request.
